# Confocal laser scanning microscopic study of the killing of metastatic colon carcinoma cells by Kupffer cells in the early onset of hepatic metastasis

**DOI:** 10.1186/1476-5926-2-S1-S50

**Published:** 2004-01-14

**Authors:** Maarten Timmers, Katrien Vekemans, David Vermijlen, Ronald De Zanger, Eddie Wisse, Filip Braet

**Affiliations:** 1 Laboratory for Cell Biology and Histology, Free University of Brussels, Laarbeeklaan 103, 1090 Brussels, Belgium; 2 Present address: Department for Molecular Biomedical Research, Molecular Cell Biology Unit, Ghent University (UGhent), Technologiepark 927, 9052 Zwijnaarde, Belgium

## Introduction

During hepatic metastasis, tumor cells are exposed to the sinusoidal environment, involving endothelial cells, Kupffer cells, pit cells (NK cells) and fat-storing cells [1]. It is known that Kupffer cells and pit cells play a direct role in killing metastasizing colon carcinoma cells [1,2]. Only few studies describe the interactions of Kupffer cells and tumor cells within the first 24 hrs of metastasis [3-6]. The importance of this early phase lies in the substantial but not complete killing of tumor cells. Also, at later stages, when different elements of host defense are involved, it is apparent that the local immune system is not capable of preventing metastasis.

Investigating rare cellular events is facilitated by studying the full depth of thick sections with confocal laser scanning microscopy. Complicated preparation and histochemical procedures and a low sampling-volume [7] characterize classical microscopic methods. In contrast, confocal laser scanning microscopy (CLSM) has the ability to study thick sections (100 micrometers), gathering 3D information and allowing the use of different fluorescent probes for different variables [8]. Localization of cells can be done by immunohistochemistry, by labeling cells *in vitro *prior to injection or by labeling them *in vivo*. The anti-metastatic function of Kupffer cells was studied by labeling tumor cells with the lipophilic probe DiO *in vitro *and Kupffer cells with fluorescent latex particles *in vivo*.

## Methods

### Animals

Male Wag/Rij rats were purchased from Harlan (The Netherlands) and were 8–12 weeks of age, weighting 240–260 g. Animals had free access to food and water. The local university ethical committee for animal welfare approved all animal experiments.

### Cultivation and Fluorescent Labeling of Cells

CC531s, a rat colon carcinoma cell line originating in Wag/Rij rats [9], was cultured in RPMI-1640 enriched with 10% fetal bovine serum, 100 micrograms/ml streptomycin, 100 U/ml penicillin and 2 mM L-glutamine under standardized conditions (37–C, 5% CO_2_). Cultured CC531s cells were enzymatically detached with trypsin (500 micrograms/ml)/EDTA (200 micrograms/ml)(reagents from Gibco Life Technologies, Ghent, Belgium). CC531s cells were resuspended to a concentration of 1 – 10^6 ^cells/ml PBS. Cells were fluorescently labeled with the lipophilic probe DiOC_18 _(D-275, Molecular Probes Inc., Eugene-Oregon, USA) as described previously [10]. Aggregates of cells were removed by sterile filtration through nylon gauze (100 mesh). Cell viability was checked by trypan blue exclusion (Gibco Life Technologies, Ghent, Belgium) and was always found to be more than 90%. Kupffer cells were marked *in vivo *by penile vein injection of 3.8 – 10^9 ^TRITC-labeled latex beads (– 2 micrometers; F-8826, Molecular Probes Inc., Eugene-Oregon, USA) resuspended in 75 Units of heparin (Leo Pharmaceutical Products, Brussels, Belgium).

### Liver Metastasis Induction

Rats were anaesthetized using Nembutal at a dose of 60 mg/100 g body weight (Leo Pharmaceutical Products, Brussels, Belgium). Early stages of liver metastasis were generated by intramesenterical injection of 1 – 10^6 ^CC531s cells suspended in 0.5 ml PBS under sterile conditions. 1, 8 and 24 hrs after injection, the liver was perfusion-fixed (9 ml/min) using 2% paraformaldehyde prepared in PBS. 100 –m sections were made and mounted on microscopic slides in Vectashield (H-1000, Vector Laboratories Inc., Burlingame, USA).

### Confocal Microscopy

Liver sections were studied by a Leica TCS SP CLSM (Leica TCS NT software version 1.6.587) equipped with Ar/HeNe lasers and installed on a Leica DM-IRBE inverted microscope [10].

## Results and Discussion

**Figure 1:**
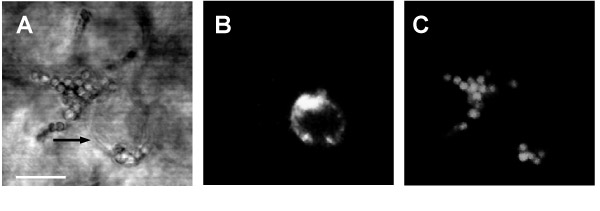
Contact between Kupffer cells and CC531s cells was observed with CLSM (A-C) and TEM (D). **A. **Bright field image illustrating the protrusions (–) from the Kupffer cell, surrounding the CC531s cell. **B. **Green fluorescent channel showing the DiO labeled CC531s cell in the same spot. **C. **Red channel showing the TRITC labeled latex particles taken up by the Kupffer cell in the same spot. Bar: 10 –m.

During the first day of metastasis, tumor cells arriving subsequently in the liver were mainly localized in the portal area's [11]. At one hour, Kupffer cells were observed in contact with tumor cells [3]. Protrusions from Kupffer cells surrounding the tumor cells could be observed using CLSM (Fig. 1) as was verified with electron microscopy [data not shown; [3]].

**Figure 2:**
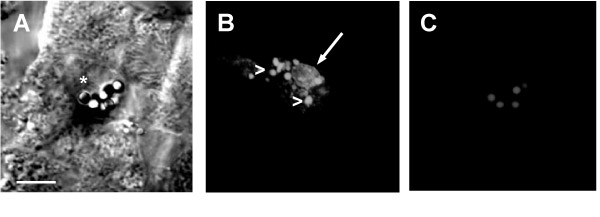
CLSM-Images of a phagocytosed CC531s cell. **A. **Bright field image of Kupffer cell (asterisk). **B. **Green fluorescent channel showing DiO-labeled cell remnants of a CC531s cell taken up by a Kupffer cell and situated in a large vacuole (arrow). Note the smaller vacuoles (>) surrounding the large vacuole. **C. **Red fluorescent channel showing TRITC-labeled latex beads (2 –m) taken up by the Kupffer cell. Bar: 10 –m.

Eight hours and 24 hrs after tumor cell administration, tumor cells phagocytosed by Kupffer cells could be frequently observed (Fig. 2). In contrast, 1 hr post injection, phagocytosed tumor cells were rarely observed, illustrating the importance of tumor cell binding prior to elimination [12]. The observation of phagocytosed tumor cells was facilitated by the presence of DiO in Kupffer cells marked by TRITC-labeled latex beads, as DiO does not transfer from labeled to unlabeled cells [10]. Large vacuoles (8 –m) could be observed surrounded by smaller vacuoles indicating the uptake of intact tumor cells [3,6,12] and their subsequent lysosomal degradation (Fig. 2) [5].

One day after the injection, non-phagocytosed tumor cells were seldom observed, showing the importance of the immune system present in the liver during this phase (first day) of metastasis [1,4-6]. These results show that CLSM is a useful tool to study the interactions occurring between Kupffer cells and tumor cells in large volumes of tissue [10].
